# Case Report: Sodium glucose cotransporter 2 inhibitor use in a pediatric patient with cancer receiving cisplatin

**DOI:** 10.3389/fped.2026.1906970

**Published:** 2026-10-30

**Authors:** Esraa Elsamny, Amr Elgehiny, Rita Swinford

**Affiliations:** 1 Department of Pediatrics, University of Texas Health Science Center at Houston, Houston, TX, United States; 2 Division of Pediatrics-Patient Care, The University of Texas MD Anderson Cancer Center, Houston, TX, United States

**Keywords:** cisplatin, cisplatin induced nephrotoxicity, nephro- protection, pediatric nephrology, pediatric oncology, SGLT2 (sodium-glucose cotransporter 2) inhibitor

## Abstract

Cisplatin is a foundation chemotherapeutic for pediatric germ cell tumors; however, its dose-dependent nephrotoxicity can limit use. Hypomagnesemia is a challenging side effect, and patients often remain hypomagnesemic despite IV and oral magnesium (Mg) supplementation. Sodium Glucose Cotransporter 2 inhibitors (SGLT2is) have been shown to mitigate cisplatin-induced nephrotoxicity (CIN) and reduce urinary Mg wasting in adults, but their use remains off-label and limited in pediatrics. We report a case of an adolescent male with metastatic germ cell tumor treated extensively with cisplatin, whose course was complicated by hypertension, hypomagnesemia, and a declining glomerular filtration rate (GFR). An SGLT2i was started three months into treatment. His GFR stabilized and his proteinuria resolved despite continued treatment with cisplatin. Additionally, he discontinued IV Mg supplementation while maintaining adequate levels on oral Mg, and his blood pressure improved with discontinuation of antihypertensives. This case highlights a potential role of SGLT2is in pediatric oncology.

## Introduction

Cisplatin and other platinum compounds have long been a cornerstone of cancer treatment in the management of solid tumors, such as ovarian, cervical and testicular cancers (1, 2). Although cisplatin has extended countless lives, some patients are left with significant renal tubular cell dysfunction, inflammation, and ischemic injury (3, 4). This is particularly important because of the kidney's role in the elimination of cisplatin from the body (5). The rate of nephrotoxicity caused by cisplatin is reported to be 28%–30% (6, 7). Cisplatin-induced nephrotoxicity (CIN) is dose-dependent and can manifest as acute kidney injury and chronic kidney disease (5). Despite cisplatin's importance, the incidence and predictors of nephrotoxicity remain poorly understood but believed to be due to a combination of mitochondrial damage, apoptosis of renal tubular cells, vasoconstriction, transporter dysfunction, and toxin accumulation (3, 6).

Hypomagnesemia is another challenging side effect (8). Patients often require aggressive supplementation to maintain adequate levels (9). Sodium-glucose co-transporter 2 inhibitors (SGLT2is) are among the newest classes of antidiabetic agents and have been shown to slow the progression of diabetic kidney disease (10, 11). Most recently, studies have demonstrated that SGLT2is attenuate cisplatin-induced nephrotoxicity and reduce urinary magnesium wasting (9, 12). To date, the use of SGLT2 inhibitors in pediatrics is limited (10).

## Case description

We report the case of a 17-year-old otherwise healthy male diagnosed with a high-risk testicular germ cell tumor metastatic to the lungs, spine, mediastinal lymph nodes, liver, and right kidney. Following an orchiectomy, he initiated chemotherapy with four cycles of bleomycin, etoposide, and cisplatin (BEP). Three months into treatment, he was seen by nephrology for elevated blood pressure, a declining glomerular filtration rate (GFR), and persistent hypomagnesemia. Despite oral magnesium protein complex supplementation, he required adjunctive intravenous magnesium which was either given in the outpatient infusion center or added to his fluids during admission.

At this point his cumulative cisplatin dose was 300 mg/m^2^. His blood pressure was 142/78 and his estimated GFR (eGFR) was 62 mL/min/1.73 m^2^ (CKiD U25) (Figure 1) (13). Dapagliflozin, an SGLT2 inhibitor (SGLT2i), was initiated at 5 mg daily, and irbesartan 150 mg daily was started. Two weeks later, due to persistent hypomagnesemia, the dapagliflozin dose was increased to 10 mg daily (Figure 1) and his irbesartan dose was increased to 300 mg daily for continued elevation in blood pressure to 155/76. He received no additional cisplatin during this 2-week interval. Due to bleomycin-induced pulmonary toxicity, his regimen was subsequently transitioned to TIP (paclitaxel, ifosfamide, and cisplatin). He received one cycle of TIP, bringing his cumulative cisplatin dose to 555 mg/m^2^.

**Figure 1:**
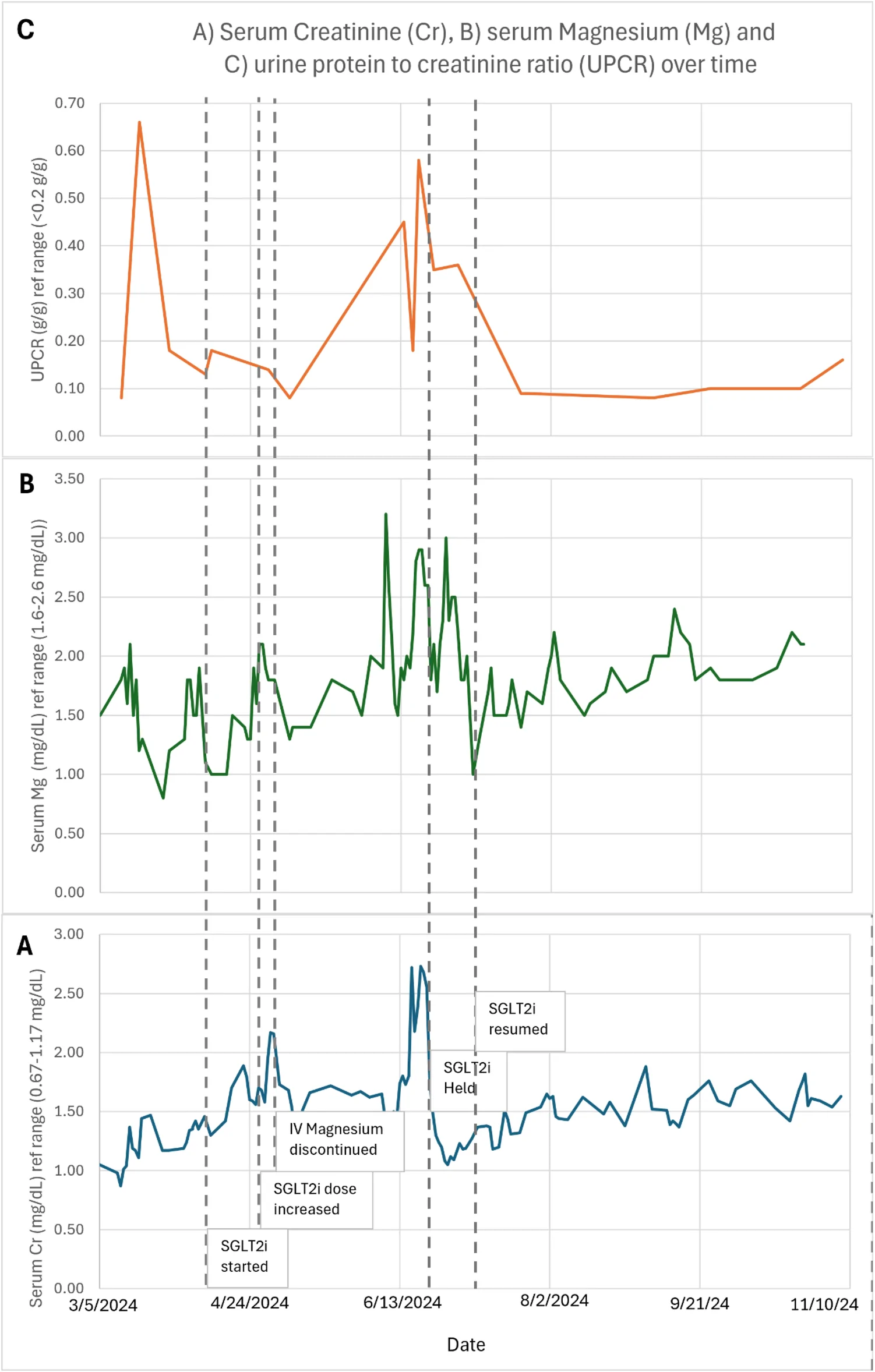
(**A**) Serum creatinine (Cr), (**B**) serum magnesium (Mg) and (**C**) urine protein to creatinine ratio (UPCR) over time.

Four months into treatment, owing to rising tumor markers, he proceeded to hematopoietic stem cell transplantation (HSCT) with carboplatin conditioning (cumulative carboplatin dose 750 mg/m^2^). His post-transplant course was complicated by septic shock and non-dialysis-requiring acute kidney injury (AKI) (Figure 1). Dapagliflozin was temporarily held during the AKI episode and resumed following renal recovery (Figure 1). All cisplatin and carboplatin doses along with hydration as per MD Anderson protocol.

His malignancy proved refractory post-HSCT, prompting a transition to an epirubicin and cisplatin regimen. He received two cycles, bringing his total cumulative cisplatin exposure to 735 mg/m^2^. Aside from the transient AKI post-HSCT, his eGFR remained stable at approximately 60 mL/min/1.73 m^2^ (CKID U25) (13). While on the Dapagliflozin, he no longer required IV magnesium supplementation and was able to maintain magnesium levels on oral magnesium alone (Figure 1). Additionally, antihypertensive therapy was fully discontinued three months after initiating the SGLT2i. Glucosuria was noted on urinalysis while on the SGLT2i.

## Discussion

Cisplatin and other platinum compounds are widely used chemotherapeutic agents for various cancers (1). Cisplatin exerts its cytotoxic effects by forming covalent bonds with purine bases on DNA strands forming DNA adducts (14). These adducts lead to the formation of intrastrand and interstrand crosslinks which distorts the DNA double helix and interferes with replication and transcription, ultimately resulting in cell cycle arrest and cell death (3, 14). Cisplatin also induces the formation of reactive oxygen species (ROS) and depletes intracellular antioxidants, leading to oxidative stress (15, 16). Additionally, it causes mitochondrial dysfunction by damaging mitochondrial DNA and generating reactive oxygen species (3, 16).

CIN is a dose-dependent side effect that often necessitates dose reduction or discontinuation, particularly because cisplatin is primarily excreted by the kidneys (6, 7). Cisplatin is taken up by the renal tubular cells via various transporters including copper transporter 1 (CTR1) (5, 17). It then accumulates inside the cells, especially proximal tubular cells, causing DNA damage, oxidative stress, and mitochondrial injury (5, 16). Vasoconstriction of renal blood vessels further contributes to renal ischemia (3, 17). CIN can present as acute kidney injury and/or chronic kidney disease, with a reported incidence of 28%–30% (6, 7). Risk is higher in patients receiving more than 50 mg/m2 body surface area (18).

Magnesium wasting is another dose-dependent adverse effect (8, 19). Among patients affected by CIN, up to 90% also experienced hypomagnesemia often necessitating aggressive supplementation to maintain normal serum levels (9). This electrolyte disturbance arises early in the course of CIN, frequently preceding elevations in serum creatinine or GFR decline (18). This is attributed to cisplatin's primary toxicity targeting the proximal convoluted tubule (PCT), with relative sparing of the glomeruli (6). Notably, magnesium supplementation has been shown to confer protective effects against CIN, even when serum magnesium levels remain within the normal range, suggesting a protective role independent of systemic magnesium concentration (20).

SGLT2is have shown promise in slowing the progression of diabetic kidney disease and other chronic kidney disease by preserving GFR (10, 11). These agents function by inhibiting glucose reabsorption in the PCT, leading to glucosuria (10, 11). Additionally, they block sodium reabsorption, resulting in natriuresis, reduced total body volume, and subsequently lower blood pressure (10, 11). Despite the attention these agents are receiving in the nephrology community, their use in pediatrics remains limited (10, 21). Adult clinical trials have demonstrated an elevated risk of genital infections associated with SGLT2 inhibitor (SGLT2i) use and an increased risk of urinary tract infections with dapagliflozin 10 mg daily (22). This potential risk, however, remains uncharacterized in pediatric populations. SGLT2i induced glucosuria creates a urinary environment favoring bacterial growth (23). In adults, these infections are predominantly lower urinary tract genital mycotic infections rather than severe UTIs or urosepsis (22). However, pediatric patients with congenital anomalies of the kidney and urinary tract (CAKUT) already carry a heightened baseline risk for UTIs due to underlying urinary obstruction, vesicoureteral reflux, or the requirement for clean intermittent catheterization (21).

Animal studies have demonstrated that cisplatin treated animals receiving SGLT2is exhibited significantly lower plasma urea and creatinine levels, as well as reduced urinary neutrophil gelatinase-associated lipocalin (NGAL) and albumin-to-creatinine ratios, compared to animals treated with cisplatin alone. These animals also showed decreased levels of inflammatory markers such as tumor necrosis factor-alpha (TNF-α) and interleukin-6 (IL-6), along with elevated antioxidant levels (15, 24). These findings suggest that SGLT2is may possess anti-inflammatory and antioxidant properties, potentially offering protective effects against CIN (15, 24). SGLT2is also regulate the expression of NKCC2, claudin -16 and TRPM6 thereby reducing magnesium wasting (19). A recent large retrospective cohort study showed that SGLT2i use mitigated CIN and cisplatin induced hypomagnesemia in diabetic patients (12). In addition, case reports have described SGLT2i use for cisplatin induced hypomagnesemia (9).

The clinical trajectory of our patient, depicted in Figure 1, demonstrates the dynamic interplay between CIN and the application of SGLT2 inhibition in an adolescent patient. Following the initiation and upward titration of dapagliflozin, a characteristic initial fluctuation in serum creatinine was observed, reflecting the acute hemodynamic alterations and reduction in intraglomerular pressure classic to this drug class (10). Remarkably, despite the severe PCT injury typically induced by heavy cisplatin exposure, the addition of dapagliflozin allowed for the successful discontinuation of all IV magnesium supplementation while maintaining adequate serum levels on oral replacement alone. This observation aligns with emerging data suggesting that SGLT2 inhibitors exert a magnesium-sparing effect, likely mediated by the downstream upregulation of specific magnesium transporters to compensate for PCT dysfunction (19).

Furthermore, the clinical utility of the drug was highlighted during the patient's SCTX which was complicated by septic shock and a profound non-dialysis-requiring AKI. As illustrated in Figure 1, holding the SGLT2i during this acute phase, followed by resumption upon clinical recovery, led to a highly stable maintenance phase. Ultimately, long-term follow-up demonstrated not only stable renal function (eGFR 60 mL/min/1.73 m^2^) and sustained suppression of proteinuria (UPC), but also a significant hemodynamic benefit that allowed for the complete cessation of high-dose antihypertensive therapy. This case illustrates a potentially durable therapeutic strategy to mitigate the chronic sequelae of CIN and manage refractory electrolyte wasting in the pediatric oncology population.

## Conclusion

This case highlights the potential role of SGLT2 inhibitors in pediatric oncology for managing cisplatin-induced nephrotoxicity and hypomagnesemia. Although concurrent irbesartan administration represents a study limitation that could have influenced renal recovery, no urinary magnesium wasting indices were collected. We hypothesize that SGLT2 inhibitor therapy stabilized renal function and mitigated magnesium loss despite extensive cisplatin exposure. This observation highlights the need for further studies to establish whether SGLT2 inhibitors provide true nephroprotection against cisplatin toxicity in children.

## Data Availability

The raw data supporting the conclusions of this article will be made available by the authors, upon request, without undue reservation.
